# Sedentary Behaviour and Telomere Length Shortening during Early Childhood: Evidence from the Multicentre Prospective INMA Cohort Study

**DOI:** 10.3390/ijerph20065134

**Published:** 2023-03-14

**Authors:** Daniel Prieto-Botella, Dries S. Martens, Desiree Valera-Gran, Mikel Subiza-Pérez, Adonina Tardón, Manuel Lozano, Maribel Casas, Mariona Bustamante, Alba Jimeno-Romero, Ana Fernández-Somoano, Sabrina Llop, Martine Vrijheid, Tim S. Nawrot, Eva-María Navarrete-Muñoz

**Affiliations:** 1 Department of Surgery and Pathology, Miguel Hernandez University, 03550 Alicante, Spain; 2 Centre for Environmental Sciences, Hasselt University, Agoralaan Gebouw D, BE-3590 Hasselt, Belgium; 3 Grupo de Investigación en Terapia Ocupacional (InTeO), Miguel Hernández University, 03550 Alicante, Spain; 4 Alicante Institute for Health and Biomedical Research (ISABIAL-FISABIO Foundation), 03010 Alicante, Spain; 5 Biodonostia Health Research Institute, Group of Environmental Epidemiology and Child Development, 20014 San Sebastian, Spain; 6 Spanish Consortium for Research on Epidemiology and Public Health (CIBERESP), 28029 Madrid, Spain; 7 Department of Clinical and Health Psychology and Research Methods, University of the Basque Country (UPV/EHU), 20018 Donostia-San Sebastián, Spain; 8 Bradford Institute for Health Research, Temple Bank House, Bradford Royal Infirmary, Duckworth Lane, Bradford BD9 6RJ, UK; 9 Unidad de Epidemiología Molecular del Cáncer, Departamento de Medicina, Instituto Universitario de Oncología del Principado de Asturias (IUOPA), Universidad de Oviedo, Julián Clavería Street s/n, 33006 Oviedo, Spain; 10 Instituto de Investigación Sanitaria del Principado de Asturias (ISPA), Roma Avenue s/n, 33001 Oviedo, Spain; 11 Epidemiology and Environmental Health Joint Research Unit, FISABIO-Universitat Jaume I-Universitat de València, 46020 Valencia, Spain; 12 Preventive Medicine and Public Health, Food Sciences, Toxicology and Forensic Medicine Department, Universitat de València, 46100 Valencia, Spain; 13 ISGlobal, Institute for Global Health, 08003 Barcelona, Spain; 14 Universitat Pompeu Fabra (UPF), 08003 Barcelona, Spain; 15 Department of Preventive Medicine and Public Health, Faculty of Medicine, University of the Basque Country (UPV/EHU), 48940 Leioa, Spain; 16 Department of Public Health & Primary Care, University of Leuven (KU Leuven), BE-3000 Leuven, Belgium

**Keywords:** lifestyle, children, genetics, screen time, epigenetics, cellular longevity

## Abstract

Sedentary behaviour (SB) may be related to telomere length (TL) attrition due to a possible pro-inflammatory effect. This study examined the association between parent-reported sedentary behaviour (SB) and leukocyte TL at the age of 4 and telomere tracking from 4 to 8 years. In the Spanish birth cohort Infancia y Medio Ambiente (INMA) project, we analysed data from children who attended follow-up visits at age 4 (n = 669) and 8 (n = 530). Multiple robust regression models were used to explore the associations between mean daily hours of SB (screen time, other sedentary activities, and total SB) at 4 years categorised into tertiles and TL at 4 years and difference in TL rank between age 4 and 8, respectively. At the age of 4, the results showed that children with the highest screen time (1.6–5.0 h/day) had a shorter TL of −3.9% (95% CI: −7.4, −0.4; *p* = 0.03) compared with children in the lowest tertile (0.0–1.0 h/day). Between 4 and 8 years, a higher screen time (highest tertile group vs. lowest tertile) was associated with a decrease in the LTL rank of −1.9% (95% CI: −3.8, −0.1; *p* = 0.03) from 4 to 8 years. Children exposed to a higher screen time at 4 years were more prone to have shorter TL at 4 and between 4 and 8 years of age. This study supports the potential negative effect of SB during childhood on cellular longevity.

## 1. Introduction

Telomeres are DNA–protein complexes located at the end of chromosomes consisting of tandem-repeated TTAGGG sequences that maintain chromosome stability and integrity [1,2]. However, due to the end replication problem of cell division, full DNA replication cannot be completed, leading to a progressive natural telomere attrition [2]. In addition, telomeres can be vulnerable to oxidative stress and systemic inflammation induced by exogenous factors that can accelerate telomere length (TL) shortening [3,4]. As a biomarker of cellular aging, [5] a reduced TL has been associated with all-cause mortality and age-related diseases, including cardiovascular pathologies [6,7,8]. Non-genetic factors that can alter the oxidative stress and inflammation balance, including environmental exposures, have been associated with shorter TL in newborns, children, and adults [9,10,11]. In recent years, several studies have shown that unhealthy lifestyles, such as sedentary behaviour (SB) in adults, can negatively affect TL [12,13,14].

SB is defined as any waking activity with an energy expenditure ≤ 1.5 metabolic equivalent of task (MET), such as watching TV or reading [15]. In children and adolescents, SB has been associated with an increased risk of obesity, cardiometabolic diseases, or psychological ill-being [16,17]. Biologically, a sedentary lifestyle could induce significant pro-inflammatory effects [18,19,20], although the underlying mechanisms still need to be clarified.

To date, no evidence for the effect of SB on TL has been shown during childhood and/or adolescence. Alternatively, few studies have examined the association, although indirectly, between physical activity (PA) and TL in young populations. The study conducted by Zhu et al. reported that vigorous PA could have a beneficial effect on TL in adolescents aged 14–18 years [21]. More recently, a randomised clinical trial in obese children aged 7–16 years showed that light PA and sedentary time were inversely associated with TL [22]. Although these results are consistent with earlier evidence for adults, the available research on TL shortening factors during childhood is still very incipient. Importantly, since TL attrition in early life has been shown as predictor of later life TL, [23] studying the environmental factors that can affect TL in paediatric populations remain crucial for understanding age-related disease in adulthood [24].

Therefore, this study had the following aims: first, to examine the cross-sectional association between parent-reported SB at the age of 4 and leukocyte TL in 4-year-old children, and second, to explore the association between parent-reported SB at 4 years and telomere tracking from 4 to 8 years. We hypothesised that higher mean daily hours of SB at age 4 would be associated with a shorter TL at the same age and a decrease in the TL rank between 4 and 8 years of age.

## 2. Materials and Methods

### 2.1. Study Population

This study was performed using data from the birth cohort study INMA (Infancia y Medio Ambiente, https://www.proyectoinma.org/). Details of the INMA study protocol have been described elsewhere [25]. Briefly, 1909 women with singleton pregnancy were recruited between 2003 and 2008 in three areas of Spain (Asturias, Gipuzkoa, and Sabadell). A sample of 1383 (72.4%) mother–child pairs were evaluated at the 4-year follow-up visit after delivery, accounting for the baseline population of the present study. In addition, a second follow-up visit was performed at 7 years in Asturias and Gipuzkoa and at 9 years in Sabadell. From now on, we refer to this second visit as the 8-year assessment. Based on the available data on the child’s SB and TL, a total of 669 children participated at the 4-year follow-up and 530 children participated at the 8-year assessment. The flowchart of the population sample included in this study is displayed in Figure S1 (Supplementary Material). This study was approved by the regional Ethical Committees and a written informed consent was obtained from all participants at each phase of the study. This study complies with the Helsinki declaration for human studies [26].

### 2.2. Parent-Reported Sedentary Behaviour

Parent-reported SB information was collected by a questionnaire based on the Children’s Leisure Activities Study Survey (CLASS) [27]. Parents were asked how many hours their child spent during weekdays and weekends watching TV/videos (screen time) and doing other sedentary activities (e.g., puzzles, books, dolls, homework, computer/videogames) outside school. Mean daily hours of screen time and other sedentary activities were calculated by averaging the time spent in these activities during weekdays and weekends as follows: (((SB time weekday × 5) + (SB time weekend × 2))/7). Once mean daily hours of screen time and other sedentary activities were estimated for each child, we calculated the total SB as the sum of these two variables. All SB variables (i.e., screen time, other sedentary activities, and total SB) were categorised into tertiles to classify the children according to low, middle, or high SB.

### 2.3. Blood Collection and DNA Extraction

Child blood samples were collected during clinical examination and properly stored in EDTA tubes. At 4 years, DNA was extracted from whole blood using the Flexigen AGKT-WB-640 (Qiagen) kit in Gipuzkoa samples, Chemagen kit (Perkin Elmer) in Sabadell, and from buffy coat applying the QIAamp DNA Mini Kit (Qiagen) in Asturias. At 8 years, DNA was extracted from buffy coat using the above-mentioned kits.

### 2.4. Leukocyte Telomere Length Measurement

Technical details on leucocyte TL measurement using qPCR [23] are described in Methods S1 (Supplementary Material). Telomeres were measured in triplicates, and on each run, a 6-point serial dilution of a pooled DNA (n = 12 DNA samples) was run to evaluate qPCR efficiency for telomere (T) and single-copy gene (S) runs. The efficiency was 107% for T runs (R^2^ ranged from 0.995 to 0.999). Leucocyte TL at 8 years in the Sabadell cohort samples was assayed previously [28] using different single-copy gene primers (see Supplementary Material for more details). Relative leucocyte TL was calculated separately for each cohort using qBase software (Biogazelle, Zwijnaarde, Belgium). In qBase, TL is calculated as a calibrated normalised relative quantity (CNRQ) [29]. The latter is achieved by first calculating the RQ based on the delta-Cq method for T and S obtained Cq values, using target specific amplification efficiencies. As the choice of a calibrator sample (sample to which subsequent normalisation is performed) strongly influences the error on the final relative quantities (as a result of the measurement error on the calibrator sample), normalisation is performed to the arithmetic mean quantification values for all analysed samples per cohort, which results in the NRQ. Finally, as samples per cohort are measured over multiple qPCR plates, 8 inter-run calibrators (IRCs) are used to calculate an additional correction factor to eliminate run-to-run differences, resulting into the final T/S ratio (CNRQ). Mathematical calculation formulas to obtain RQ, NRQ, and CNRQs are provided by Hellemans et al., 2007 [29]. On each run, the reliability/accuracy of the applied protocol was assessed by calculating the intraclass correlation coefficients (ICC) of triplicate measures for T values (0.957; 95% CI: 0.954–0.96; *p* < 0.0001), S values (0.968; 95% CI: 0.965–0.97; *p* < 0.0001), and T/S ratio’s (0.925; 95% CI: 0.918–0.93; *p* < 0.0001), using the ICC R-code provided by the Telomere Research Network [30]. In addition, based on the 8 IRCs ran over all the qPCR plates, an inter-assay ICC was calculated (0.898; 95% CI: 0.77–0.948; *p* < 0.0001). Based on the standard curves, qPCR efficiency for T runs was 107 on average.

### 2.5. Study Covariates

Covariates included data collected during pregnancy or at birth: child’s sex (male or female), cohort (Asturias, Gipuzkoa, or Sabadell), preterm birth (no or yes), mother’s periconceptional body mass index (BMI, calculated as weight in kilograms (kg) divided by height in meters squared (m^2^)), mother’s country of origin (Spain or other) and mother’s educational level (primary or less, secondary school, or university). At the 4 years follow-up interview, the following was collected: child’s characteristics (age (years)), BMI (kg/m^2^), blood extraction date, season of blood extraction (spring, summer, autumn, or winter), total energy intake (in kilocalories (kcal) per day), ultra-processed food intake according to the NOVA classification (grams (g) per day) [31], relative Mediterranean diet (rMed) score [32], extracurricular PA (MET-hours per day) [33], and mother’s characteristics (age (years) and smoking status (yes or no)). Child nutritional data were assessed using a food frequency questionnaire previously validated in Spanish children [34]. Follow-up time was defined as the interval between the child’s age at the baseline and age at the visit assessment at 8 years.

### 2.6. Statistical Analysis

R software version 4.1.0 (R Core Team. R: A language and environment for statistical computing. R Foundation for Statistical Computing, Vienna, Austria; http://www.R-project.org) was used to conduct the statistical analyses. All applied statistical tests were bilateral, and the significance level was established at 0.05. Distribution of the continuous variables was checked using the Kolmogorov–Smirnov test. Mother and child characteristics were described according to the children’s total SB time tertiles and compared using the ANOVA, Kruskal–Wallis, and Chi-square tests.

Multiple robust linear regression models using the robustbase R package [35] were conducted separately to explore the associations with TL at 4 years and changes in TL ranking from 4 to 8 years. To control potential confounding, several models were fitted by including all covariates with *p* < 0.2 in the bivariate analysis and those that changed the magnitude of main effect by 10% after a backward–forward elimination procedure [36]. In the cross-sectional analysis at 4 years of age, the association between SB and TL was explored using log-level regression models, where TL was log10-transformed. Three different models were fitted for this analysis: Model 1 was adjusted for blood storage date and cohort; Model 2 included variables of Model 1 plus child’s daily total energy intake, daily ultra-processed food intake, and age at baseline; and Model 3 included variables of Model 2 plus child’s sex. Final estimates were presented as percentage (%) change in TL. Since TL at 8 years in children from Sabadell was measured using different single-copy genes, the values of TL at baseline (i.e., 4 years) were not directly comparable to those at follow-up (i.e., 8 years). Therefore, to evaluate the association between SB at 4 years and changes in TL from 4 to 8 years, we used a ranking method for examining telomere tracking [37]. Firstly, we ranked TL at 4 and 8 years separately by cohort from the longest to the shortest value (coded from 1 to n). The Spearman’s correlation between 4- and 8-year TL ranking was 0.58 (*p* < 0.001). Secondly, we calculated the difference in telomere rank for each child between periods. The following formula was used to calculate the difference in telomere ranking (Δ*R*):

(1) $$\Delta R=\frac{\left(R_{1}-R_{2}\right)}{\left(\frac{n-1}{100}\right)}\;$$

where *n* is the sample size, *R*_1_ is the baseline rank, and *R*_2_ is the follow-up rank. Due to the difference in telomere ranking, which is directly determined by the sample size, we transformed it into a value of 100 to ease the interpretation in percentage terms. We explored the association with TL ranking using four different models. Models 1, 2, and 3 were equally adjusted for the variables used in the cross-sectional analysis. Model 4 was additionally adjusted for the follow-up time and TL at baseline (i.e., 4 years). Final estimates were provided as % change in ranking, where a negative value indicated a decline in telomere ranking as a relative change between TL at 4 years and 8 years.

To quantify the heterogeneity among the study cohorts, all associations were initially analysed using meta-analytic techniques to obtain combined estimates. The heterogeneity was quantified using I^2^ statistics [36,38] with the meta R package [39]. Since all I^2^ values obtained for the main outcomes were < 50%, we performed the analysis adding the cohort variable to the adjustment of all the models.

Several sensitivity analyses were also conducted to examine the robustness of the main findings. Using Model 3 and Model 4 as the main models for the association with TL at 4 years and with TL ranking from 4 to 8 years, respectively, we separately explored the effect of the following child variables: other sedentary activities, rMed score, BMI, season of blood extraction, and extracurricular PA. In addition, mother variables such as periconceptional BMI, smoking status at baseline, and educational level were added jointly. We also examined whether the associations changed substantially according to the child’s sex. Finally, we conducted an analysis excluding children born preterm.

## 3. Results

### 3.1. Characteristics of the Study Population

Table 1 describes the general characteristics of the study population by the total SB time tertiles. A total of 350 children (52.3%) were boys and the median (IQR) of BMI was 16.0 (15.2–17.0) kg/m^2^. The mean (SD) of age was 4.4 (0.2) and 8.5 (0.6) years at baseline and follow-up, respectively. Overall, children spent a total of 2.6 (2.0–3.6) h/day in SB. Children located in the highest total SB tertile showed higher daily energy (1616 kcal.; 95% CI: 1453, 1862) and UPF (417 g; 95% CI: 291, 576) intakes. In addition, children grouped into the middle and high tertiles of total SB presented lower extracurricular PA (9.3 MET-h/day; 95% CI: 6.6, 12.4 and 9.5 MET-h/day; 95% CI: 6.5, 12.5, respectively) compared with the lowest tertile. Regarding maternal characteristics, women had a mean age at baseline of 37.1 (4.2) years, and a median (IQR) periconceptional BMI of 22.9 (20.9–25.5) kg/m^2^. The vast majority were mothers born in Spain (94.0%) and 39.6% had studied at university. However, mothers whose children were in the highest total SB time tertile had greater rates of primary or secondary studies compared to those whose children were in the other tertiles. The comparison of mother and child characteristics before and after participant selection is provided in Table S1 (Supplementary Materials).

### 3.2. Sedentary Behaviour and Telomere Length at the Age of 4

In both minimal adjusted and fully adjusted models, daily screen time was negatively associated with TL in children at the age of 4 (Table 2). Compared to children with lower daily screen time, those who spent from 1.1 to 1.5 hr/day and from 1.6 to 5.0 h/day watching TV/videos showed a shorter TL (−3.3; 95% CI: −6.7, 0.4; *p* = 0.07 and −3.9; 95% CI: −7.4, −0.4; *p* = 0.03, respectively). Estimates for children in the middle tertile of other sedentary activities and those with middle and high total SB did not reach statistical significance.

### 3.3. Association between Sedentary Behaviour at 4 Years and Telomere Length Ranking from 4 to 8 Years

The association between SB variables at the age of 4 and telomere ranking from 4 to 8 years is shown in Table 2. An increased screen time was generally associated with a reduction in the TL ranking between 4 and 8 years of age, although the main results were observed after applying the fully adjusted model including the relevant variables besides the follow-up time and TL at baseline (Model 4). Children in the highest tertile of daily screen time presented a downward (accelerated shortening) shift in TL ranking of −1.9% (95% CI: −3.8, −0.1; *p* = 0.03) compared to those situated in the lowest tertile. No association was observed for other sedentary activities and total SB.

### 3.4. Sensitivity Analysis

**Figure 1:**
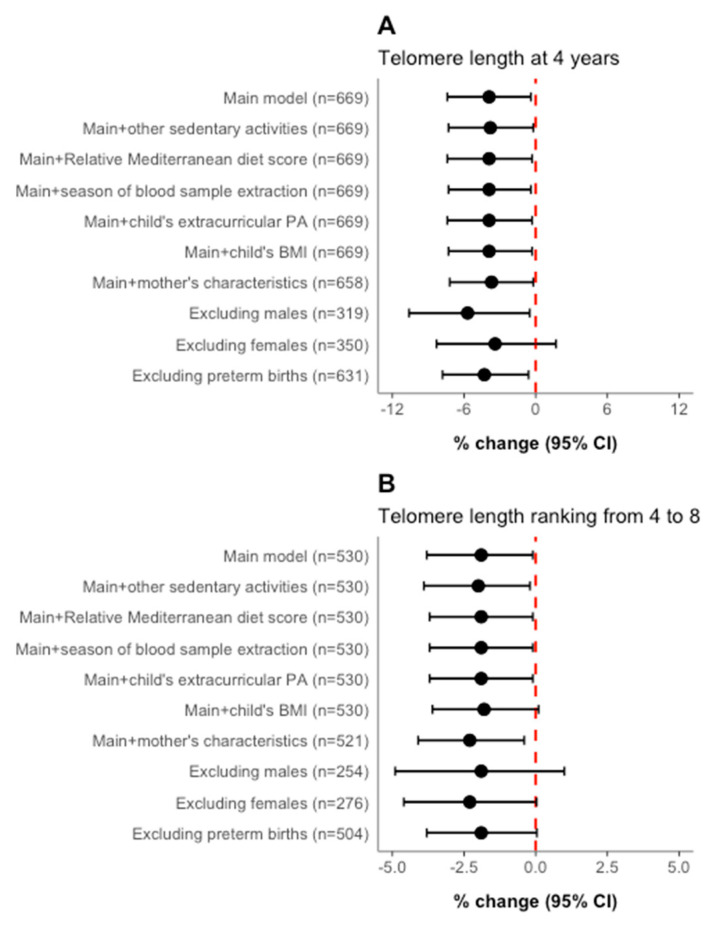
Sensitivity analyses for the associations between high screen time and telomere length at 4 years (**A**) and telomere length ranking from 4 to 8 years (**B**). Abbreviations: BMI, body mass index; PA, physical activity. CI, confidence interval. Adjustment variables: other sedentary activities (low, middle, and high); relative Mediterranean diet score (rMed, continuous), season of blood sample extraction (spring, summer, autumn, or winter); child’s extracurricular PA (METs-h/day) and child’s BMI (kg/m^2^). Mother characteristics include the following: periconceptional BMI (kg/m^2^); smoking status at baseline (yes or no), and educational level (primary, secondary, or university). (**A**): Main model adjusted for blood storage time, cohort (Asturias, Gipuzkoa, and Sabadell), child’s age (years), sex (male or female), energy intake (kcal/day), and ultra-processed food intake (g/day). The associations were estimated as % change in TL compared to the reference group (lowest tertile). (**B**): Main model adjusted for blood storage time, cohort (Asturias, Gipuzkoa, and Sabadell), child’s age (years), sex (male or female), energy intake (Kcal/day), ultra-processed food intake (g/day), follow-up time (years), and baseline leucocyte telomere length. The estimates were expressed as % change in TL ranking compared with the reference group (lowest tertile).

Figure 1 shows the sensitivity analyses for the associations between high daily screen time and TL at 4 years and TL ranking from 4 to 8 years. The association of high screen time with TL at 4 years increased substantially when excluding the boys (−5.7; 95% CI: −10.6, −0.5), although it slightly dropped when excluding the girls (−3.4; 95% CI: −8.3, 1.7). However, we found no statistically significant interaction term between screen time vs. child’s sex (*p* ≥ 0.2). The effect of high screen time on TL ranking from the age of 4 to 8 was slightly reinforced when adjusting for mother’s characteristics (−2.3; 95% CI: −4.1, −0.4) and when excluding girls (−2.3; 95% CI: −4.6, 0.0). Our findings remained robust after excluding or adjusting for other relevant potential variables.

## 4. Discussion

This study supports the fact that a higher screen time at 4 years is associated with a shorter TL at the same age and with a reduction in telomere ranking between the ages of 4 and 8. Although we observed that a general sedentary lifestyle also tended to have a negative effect on TL, the main findings disclosed that TL attrition during childhood was mainly due to a higher screen time after adjusting for relevant variables such as a child’s BMI or extracurricular PA. Our findings are consistent with previous studies conducted in adults and, to our knowledge, this is the first time that this association has been reported at early ages. Moreover, it should be noted that the analysis for the TL ranking may suppose valuable evidence to reinforce the limited results obtained from the research on the child population.

To date, few studies have explored the relationship between lifestyle factors such as SB, PA, or TL in youth. A cross-sectional study conducted in 667 adolescents aged 14–18 years showed a positive association between vigorous PA and TL [21]. Similarly, a recent randomised clinical trial of a lifestyle intervention in 102 Spanish children (7–16 years) with abdominal obesity indicated that higher levels of PA were positively associated with TL, whereas SB and light PA showed a negative effect [22]. Based on these previous results, in this study, we examined whether a child’s PA or BMI could be likely confounders of the detrimental effect of SB that we observed on TL. After accounting for these covariates, the estimates did not show changes, suggesting that the effect found for screen time was independent of them.

Several studies have suggested that a sedentary lifestyle could increase the concentration of pro-inflammatory cytokines and adipokines, incrementing chronic low-grade inflammation [13,19]. Although the underlying biological mechanism for the association between SB and TL shortening still needs to be clarified, a potential explanation may be attributed to the fact that a low-grade inflammatory state may affect telomere homeostasis, thus inducing TL shortening via oxidative stress [40]. Importantly, SB has been associated with the rise of pro-inflammatory biomarkers such as C-Reactive Protein (CRP), leptin, and interleuckin-6 in children aged 6–8 years [41,42]. In addition, higher TV viewing time in 7–10-year-old children has been associated with greater levels of CRP and of sVCAM-1, a biomarker of endothelial dysfunction [43]. Biologically, the increment of pro-inflammatory biomarkers may induce an increase in apoptosis, cellular senescence, and oxidative stress, augmenting systemic inflammation and cellular aging, which are subsequently linked with TL shortening [44].

The results of this study are in accordance with cross-sectional studies conducted in the adult population. A study published by Xue and colleagues with 518 participants (20–70 years) showed that every hour/day spent watching TV was associated with an TL shortening of 72 base pairs [12]. In fact, results from the same study indicated that adults aged 20 to 40 in the highest tertile of daily screen time had a 4.0% shorter TL, which is similar to the results obtained in the present study (3.9% reduction in TL). Another study with 6405 adults (20–84 years) from the 1999–2002 National Health and Nutrition Examination Survey (NHANES) observed that for every 1 h/day of screen-based SB, participants had 7% increased odds of having TL in the lowest tertile [13]. However, a later study based on the same data did not find an association between SB and TL [45]. In sum, although the available evidence seems to indicate that SB has a negative effect on TL, the results remain inconsistent, probably as a consequence of the different criteria used for the measurement of SB as well as resulting from other factors. More research is therefore needed to clarify the role of SB on cellular longevity.

Our study presents several strengths. First, the prospective design of the INMA project allowed us to verify the negative effects of SB on TL attrition observed at the age of 4 in a later assessment during childhood. Second, we included different types of SB that we examined as independent variables to account for possible differences in sedentary lifestyles, which may provide more support to the effect found for the screen time. Third, the main associations remained similar after applying the sensitivity analyses, reinforcing the findings’ robustness. Finally, it should be noted that the study population size was considerably reduced after participant selection. However, there were no differences between the included and non-included participants, suggesting that a selection bias did not occur (Table S1 in the Supplementary Material).

However, our study should be interpreted within the context of its potential limitations. Our SB variables were assessed using a questionnaire based on the CLASS, which has not been validated in Spanish children. In this sense, although a potential misclassification may occur, it should be nondifferential. Regarding TL, different DNA extraction kits were used among cohorts. Additionally, TL at 8 years from the Sabadell cohort was measured with a slightly different qPCR methodology. Nevertheless, we minimised the impact of these variations by normalising the TL by cohort and using a ranking approach in the analysis.

## 5. Conclusions

To our knowledge, this is the first study to report a negative association between screen time and TL during childhood. Importantly, the negative effect of a higher screen time on TL observed at 4 years was prospectively confirmed in a reduction in telomere tracking from 4 to 8 years. This study supports the potential adverse effect of SB on human health and stresses the need for further prospective studies to confirm these results.

## Figures and Tables

**Table 1:** Characteristics of mother–child pairs from the INMA study according to total sedentary time tertiles (h/day) at the age of 4 years.

Characteristics	Total	Tertiles of Total Sedentary Time			*p* ^a^
		Low (0.0–2.2)	Middle (2.3–3.1)	High (3.2–7.9)	
Number of participants, n (%)	669 (100)	223 (33.3)	226 (33.8)	220 (32.9)	
Child’s characteristics at baseline ^b^					
Age, years, mean (SD)	4.4 (0.2)	4.4 (0.2)	4.4 (0.2)	4.4 (0.2)	0.69
Age at follow-up, years, mean (SD) ^c^	8.5 (0.6)	8.3 (0.6)	8.5 (0.6)	8.6 (0.7)	0.001
Sex, n (%)					0.31
Male	350 (52.3)	122 (54.7)	109 (48.2)	119 (54.1)	
Female	319 (47.7)	101 (45.3)	117 (51.8)	101 (45.9)	
Preterm birth, yes, n (%) ^d^	30 (4.5)	7 (3.2)	13 (5.8)	10 (4.6)	0.41
BMI, kg/m^2^	16.0 (15.2–17.0)	15.9 (15.1–16.9)	16.0 (15.4–17.1)	16.1 (15.2–17.0)	0.61
Energy intake, kcal/day	1583 (1390–1820)	1542 (1345–1751)	1593 (1394–1846)	1616 (1453–1862)	0.001
UPF intake, g/day	381 (279–554)	374 (269–516)	379 (280–563)	417 (291–576)	0.05
rMed score	9.0 (7.0–10.0)	9.0 (7.0–11.0)	9.0 (7.0–10.0)	8.0 (7.0–10.0)	0.16
Extracurricular PA, MET-h/day	9.7 (6.8–12.9)	10.0 (7.5–13.5)	9.3 (6.6–12.4)	9.5 (6.5–12.5)	0.02
Cohort, n (%)					< 0.001
Asturias	273 (40.8)	82 (36.8)	104 (46.0)	87 (39.5)	
Gipuzkoa	131 (19.6)	66 (29.6)	42 (18.6)	23 (10.5)	
Sabadell	265 (39.6)	75 (33.6)	80 (35.4)	110 (50.0)	
Season of blood extraction, n (%)					0.21
Winter	165 (24.7)	49 (22.0)	62 (27.4)	54 (24.5)	
Spring	177 (26.5)	56 (25.1)	64 (28.3)	57 (25.9)	
Summer	180 (26.9)	71 (31.8)	58 (25.7)	51 (23.2)	
Autumn	147 (21.9)	47 (21.1)	42 (18.6)	58 (26.4)	
Mothers’ characteristics					
Age at baseline, years, mean (SD)	37.1 (4.2)	37.4 (4.0)	36.9 (4.0)	37.0 (4.4)	0.35
Country of origin, Spain, n (%) ^e^	626 (94.0)	212 (95.5)	209 (92.9)	205 (93.6)	0.48
Periconceptional BMI, kg/m^2^	22.9 (20.9–25.5)	22.3 (20.6–25.1)	23.2 (21.2–25.4)	22.9 (21.0–26.2)	0.07
Smoking status, n (%) ^d^					0.59
Yes	161 (24.1)	50 (22.7)	53 (23.7)	58 (26.7)	
No	500 (74.7)	170 (77.3)	171 (76.3)	159 (73.3)	
Educational level, n (%) ^e^					0.005
University	265 (39.6)	104 (46.6)	95 (42.0)	66 (30.4)	
Secondary	270 (40.4)	85 (38.1)	89 (39.4)	96 (44.2)	
Primary	131 (19.6)	34 (15.3)	42 (18.6)	55 (25.4)	

Abbreviations: h, hour; SD, standard deviation; BMI, body mass index; UPF, ultra-processed food; rMed, relative Mediterranean Diet; PA, physical activity; MET, metabolic equivalent of task. ^a^ Chi-squared, Kruskall–Wallis, or ANOVA tests across tertiles of total sedentary time; ^b^ Child’s and mother’s characteristics presented as median (IQR, interquartile range) unless otherwise indicated. ^c^ Data available for n = 530. ^d^ Data available for n = 661. ^e^ Data available for n = 666.

**Table 2:** Association between sedentary behaviour in tertiles (h/day) and telomere length at the age of 4 and changes in telomere ranking from 4 to 8 years.

Sedentary Behaviour	n	Model 1 ^a^		Model 2 ^b^		Model 3 ^c^		Model 4 ^d^	
		% Change (95% CI)	*p*	% Change (95% CI)	*p*	% Change (95% CI)	*p*	% Change (95% CI)	*p*
Association for TL at 4 years (n = 669) ^e^									
Screen time									
Low (0.0–1.0)	271	Ref		Ref		Ref			
Middle (1.1–1.5)	184	−3.4 (−6.9, 0.1)	0.05	−3.7 (−7.1, −0.1)	0.04	−3.3 (−6.7, 0.4)	0.07	-	-
High (1.6–5.0)	214	−4.3 (−7.6, −0.9)	0.01	−4.6 (−7.9, −1.1)	0.01	−3.9 (−7.4, −0.4)	0.03	-	-
Other sedentary activities									
Low (0.0–1.0)	278	Ref		Ref		Ref			
Middle (1.1–1.5)	190	−2.1 (−5.5, 1.4)	0.22	−2.0 (−5.4, 1.5)	0.26	−2.4 (−5.8, 1.1)	0.17	-	-
High (1.6–4.4)	201	1.6 (−2.0, 5.3)	0.38	1.8 (−1.8, 5.5)	0.34	1.1 (−2.4, 4.8)	0.53	-	-
Total sedentary behaviour									
Low (0.0–2.2)	223	Ref		Ref		Ref			
Middle (2.3–3.1)	226	−2.1 (−5.7, 1.7)	0.27	−2.1 (−5.7, 1.7)	0.28	−2.4 (−6.0, 1.4)	0.20	-	-
High (3.2–7.9)	220	−2.2 (−5.7, 1.4)	0.22	−2.1 (−5.6, 1.5)	0.24	−2.1 (−5.6, 1.5)	0.25	-	-
Association for TL ranking form 4 to 8 years (n = 530) ^f^									
Screen time									
Low (0.0–1.0)	219	Ref		Ref		Ref		Ref	
Middle (1.1–1.5)	143	−0.4 (−2.4, 1.5)	0.65	−0.3 (−2.2, 1.6)	0.76	−0.4 (−2.3, 1.6)	0.71	−1.0 (−2.8, 0.9)	0.30
High (1.6–5.0)	168	−1.6 (−3.5, 0.3)	0.10	−1.3 (−3.2, 0.6)	0.18	−1.3 (−3.3, 0.6)	0.16	−1.9 (−3.8, −0.1)	0.03
Other sedentary activities									
Low (0.0–1.0)	227	Ref		Ref		Ref		Ref	
Middle (1.1–1.5)	152	0.7 (−1.1, 2.5)	0.43	0.7 (−1.0, 2.5)	0.41	0.8 (−1.0, 2.6)	0.37	0.7 (−1.1, 2.4)	0.45
High (1.6–4.4)	151	0.7 (−1.4, 2.8)	0.52	0.7 (−1.4, 2.8)	0.51	0.8 (−1.3, 2.9)	0.47	1.4 (−0.6, 3.3)	0.17
Total sedentary behaviour									
Low (0.0–2.2)	177	Ref		Ref		Ref		Ref	
Middle (2.3–3.1)	177	0.1 (−1.7, 2.0)	0.88	0.2 (−1.7, 2.1)	0.81	0.2 (1.6, 2.1)	0.80	−0.2 (−2.0, 1.6)	0.82
High (3.2–7.9)	176	0.5 (−1.5, 2.4)	0.63	0.6 (−1.4, 2.5)	0.56	0.6 (−1.4, 2.5)	0.57	0.2 (−1.6, 2.1)	0.79

^a^ Model 1: model adjusted for blood storage date and cohort (Asturias, Gipuzkoa, and Sabadell); ^b^ Model 2: Model 1+ additional adjustment for child’s total energy intake (kcal./day), ultra-processed food intake (g/day), and age at baseline (years). ^c^ Model 3: Model 2+ additional adjustment for child’s sex (male or female). ^d^ Model 4: Model 3+ additional adjustment for follow-up time (years) and baseline leucocyte telomere length. ^e^ The associations for TL at 4 years were estimated as % change in leucocyte telomere length compared to the reference group of sedentary behaviour (lowest tertile); ^f^ The associations for TL ranking from 4 to 8 years were estimated as % change in ranking, a negative difference in ranking means a decline in ranking compared with the reference group of sedentary behaviour (lowest tertile).

## Data Availability

Not applicable.

## Supplementary Materials

The following supporting information can be downloaded at: https://www.mdpi.com/article/10.3390/ijerph20065134/s1. Methods S1: Average relative telomere length measurement using qPCR; Table S1: Comparison of mother–child pairs characteristics before and after participant selection for cross-sectional and longitudinal analyses; Figure S1: Flow chart of participant selection; Figure S2: Distribution plot of telomere length in the study participants. Reference [46] is cited in Supplementary Materials.
