# Supplementary material for: Sedentary Behaviour and Telomere Length Shortening during Early Childhood: Evidence from the Multicentre Prospective INMA Cohort Study
Source: Int J Environ Res Public Health. 2023 Mar 14;20(6):5134. doi: 10.3390/ijerph20065134 (PMC10048855; doi:10.3390/ijerph20065134)
Supplement: Supplementary file 1 [file ijerph-20-05134-s001.zip › ijerph-2173698-supplementary.pdf]

Supplementary Material

Methods S1. Average relative telomere length measurement using qPCR.

Table S1. Comparison of mother–child pair characteristics before and after participant selection for cross-sectional and longitudinal analyses.

Figure S1. Flow chart of participant selection.

Figure S2. Distribution plot of telomere length in the study participants.

References.

This supplemental material has been provided by the authors to give readers additional information about their work.

Methods S1. Average relative telomere length measurement using qPCR.

Average relative telomere length was measured using a modified qPCR protocol in accordance with Cawthon et al. [1] that was reported by Martens and colleagues [2]. Firstly, DNA quantity and purity was assessed using a Nanodrop 1000 spectrophotometer (Isogen, Life Science, Belgium) considering the DNA pure when the A260/280 was greater than 1.80 and A260/230 greater than 2.0. DNA integrity was assessed by agarose gel-electrophoresis. To ensure a uniform DNA input of 5 ng for each qPCR reaction, samples were diluted and checked using the Quant-iT™ PicoGreen® dsDNA Assay Kit (Life Technologies, Europe). All samples were measured in triplicates using a 7900HT Fast Real-time PCR System (Applied Biosystems) in a 384-well format. The reaction mixture used to assess telomeres contained 1x QuantiTect SYBR Green PCR master mix (Qiagen, Inc., Venlo, the Netherlands), 2 mM dithiothreitol (DTT), 300 nM telg primer (ACACTAAGGTTTGGGTTTGGGTTTGGGTTTGGGT TAGTGT), and 900 nM telc primer (TGTTAGGTATCCCTATCCCTATCCCTATCCCTATCCCTAACA). The applied cycling conditions were as follows: 1 cycle at 95°C for 10 min, 2 cycles at 94°C for 15 sec and 49°C for 2 min, and 30 cycles at 94°C for 15 sec, 62°C for 20 sec, and 74°C for 1 min and 40 sec. The single-copy gene (human β globin) qPCR mixture contained 1x QuantiTect SYBR Green PCR master mix, 400 nM HBG1 primer (GCTTCTGACACAACTGTGTTCACTAGC), and 400 nM HBG2 primer (CACCAACTTCATCCACGTTCACC). The single-copy gene used in Sabadell samples at 8 years was different and contained 1x QuantiTect SYBR Green PCR master mix, 300 nM 36B4u primer (CAGCAAGTGGGAAGGTGTAATCC), and 500 nM 36B4d primer (CCCATTCTATCATCAACGG GTACAA) [3]. The same cycling conditions were used: 1 cycle at 95°C for 10 min, 40 cycles at 95°C for 15 sec, and 58°C for 1 min and 20 sec. After PCR cycling, individual qPCR curves and melt curves were visually inspected and when a run error was observed the Cq value was removed from subsequent analysis. In addition, when triplicate measures showed a deviation of more than 0.3 in Cq values, these were removed from subsequent analysis. In total, n= 6 samples did not meet these criteria.

Table S1. Comparison of mother–child pair characteristics before and after participant selection for cross-sectional and longitudinal analyses.

Table S1. Comparison of mother–child pair characteristics before and after participant selection for analyses at 4 years and 8 years.

Characteristic	Time [a]		
	**Eligible at 4 years**	**Available at 4 years**	**Available at 8 years**
Number of participants	1383	669	530
**Child's characteristics at 4 years [b]**			
Age, years, mean (SD)	4.4 (0.2)	4.4 (0.2)	4.4 (0.2)
Sex, n (%)			
Male	713 (51.6)	350 (52.3)	276 (52.1)
Female	670 (48.4)	319 (47.7)	254 (47.9)
BMI, Kg/m^2	16.0 (15.2 to 17.0)	16.0 (15.2 to 17.0)	16.0 (15.3 to 16.9)
Missing, n (%)	5 (0.4)	0 (0.0)	0 (0.0)
Energy intake, kcal/day	1556 (1343 to 1793)	1583 (1390 to 1820)	1563 (1377 to 1798)
Missing, n (%)	68 (4.9)	0 (0.0)	0 (0.0)
UPF intake, g/day	397 (284 to 552)	381 (279 to 554)	380 (277 to 552)
Missing, n (%)	68 (4.9)	0 (0.0)	0 (0.0)
Fruits and vegetables intake, g/day	207 (145 to 295)	219 (155 to 304)	216 (153 to 305)
Missing, n (%)	68 (4.9)	0 (0.0)	0 (0.0)
Relative Mediterranean diet score	8.0 (7.0 to 10.0)	9.0 (7.0 to 10.0)	9.0 (7.0 to 11.0)
Missing, n (%)	68 (4.9)	0 (0.0)	0 (0.0)
Extracurricular PA, MET-hr/day	9.9 (7.0 to 13.1)	9.7 (6.8 to 12.9)	9.8 (7.1 to 12.9)
Missing, n (%)	38 (2.7)	0 (0.0)	0 (0.0)
Cohort, n (%)			
Asturias	412 (29.8)	273 (40.8)[c]	194 (36.6)[c]
Gipuzkoa	412 (29.8)	131 (19.6)	123 (23.2)
Sabadell	559 (40.4)	265 (39.6)	213 (40.2)
Season of blood extraction, n (%)			
Winter	221 (16.0)	165 (24.7)	130 (24.5)
Spring	287 (20.8)	177 (26.5)	133 (25.1)
Summer	313 (22.6)	180 (26.9)	155 (29.2)
Autumn	264 (19.1)	147 (21.9)	112 (21.2)
Missing	298 (21.5)	0 (0.0)	0 (0.0)
**Mothers' Characteristics at 4 years**			
Age, years, mean (SD)	36.7 (4.0)	37.1 (4.2)	37.2 (4.0)
Missing, n (%)	81 (5.9)	0 (0.0)	0 (0.0)
Country of origin, Spain, n (%)	1298 (94.4)	626 (94.0)	497 (94.0)
Missing, n (%)	8 (0.6)	3 (0.4)	0 (0.0)
Periconceptional BMI, kg/m^2	22.7 (20.8 to 25.2)	22.9 (20.9 to 25.5)	22.9 (20.8 to 25.3)
Missing, n (%)	13 (0.9)	0 (0.0)	0 (0.0)
Smoking status, n (%)			
Yes	314 (22.7)	161 (24.1)	120 (22.6)
No	1024 (74.0)	500 (74.7)	404 (76.2)
Missing	45 (3.3)	8 (1.2)	6 (1.2)
Educational level, n (%)			
University	557 (40.3)	265 (39.6)	218 (41.1)
Secondary	562 (40.6)	270 (40.4)	211 (39.8)
Primary	246 (17.8)	131 (19.6)	98 (18.5)
Missing	18 (1.3)	3 (0.4)	3 (0.6)

Abbreviations: hr, hour; SD, standard deviation; BMI, body mass index; UPF, ultra-processed food; PA, physical activity; MET, metabolic equivalent of task.

[a] Differences between sample size available at 4 years vs. eligible at 4 years and available at 8 years vs. available at 4 years were assessed using ANOVA and chi-squared tests.

[b] Child's and mother's characteristics presented as median (IQR, interquartile range) unless otherwise indicated.

[c] P value < 0.05 vs. participants eligible at 4 years.

Figure S1. Flow chart of participant selection.

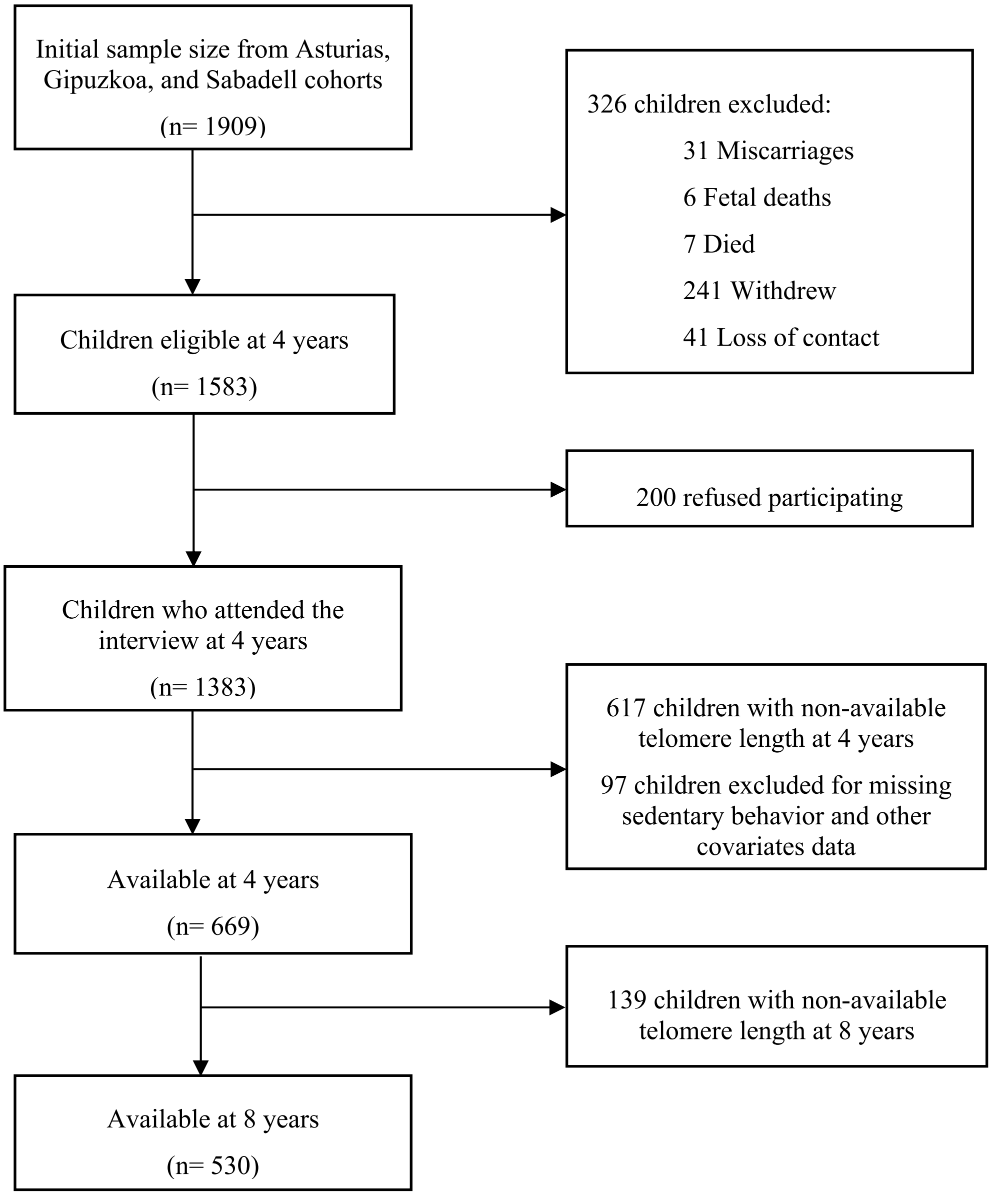

Figure S2. Distribution plot of telomere length in the study participants

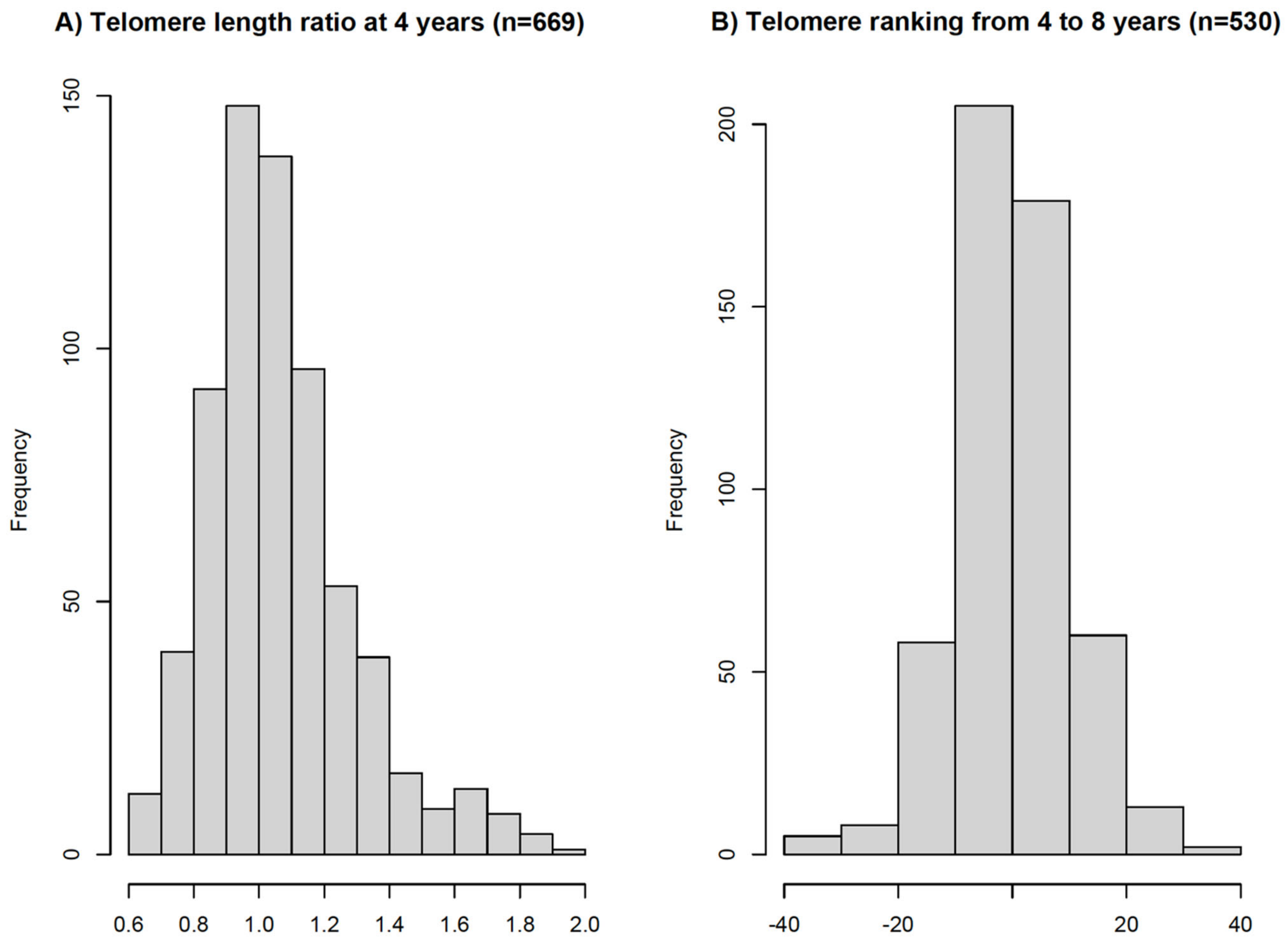

References

1. Cawthon RM. Telomere length measurement by a novel monochrome multiplex quantitative PCR method. Nucleic Acids Res. (2009)37(3):e21. doi:10.1093/nar/gkn1027

2. Martens DS, Van Der Stukken C, Derom C, Thiery E, Bijnens EM, Nawrot TS. Newborn telomere length predicts later life telomere length: Tracking telomere length from birth to child- and adulthood. EBioMedicine. (2021)63:103164. doi:10.1016/j.ebiom.2020.103164

3. Martens DS, Janssen BG, Bijnens EM et al. Association of Parental Socioeconomic Status and Newborn Telomere Length. JAMA Netw Open. (2020)3(5):e204057. doi:10.1001/jamanetworkopen.2020.4057
